# The macrophage sterol transport protein ORP2 promotes cholesterol efflux and prevents foam cell formation and atherosclerosis

**DOI:** 10.1016/j.jbc.2025.110228

**Published:** 2025-05-09

**Authors:** Xiaowei Wang, Kenan Peng, Yudi Zhao, Liwen Qiu, Chenxi Liang, Yaqian Dou, Qianqian Dong, Xiaoting Ma, Jinye Tang, Yidan Ma, Lin Liu, Mingqi Zheng, Hongyuan Yang, Mingming Gao

**Affiliations:** 1 Laboratory of Lipid Metabolism, Department of Biochemistry and Molecular Biology, The Key Laboratory of Neural and Vascular Biology, Ministry of Education, The Key Laboratory of Vascular Biology of Hebei Province, Cardiovascular Medical Science Center, Hebei Medical University, Shijiazhuang, Hebei, China; 2 Laboratory Department of Hebei General Hospital, Shijiazhuang, Hebei, China; 3 Department of Clinical Laboratory, The Second Hospital of Hebei Medical University, Shijiazhuang, Hebei, China; 4 Department of Biochemistry and Molecular Biology, College of Basic Medicine, Hebei University of Chinese Medicine, Shijiazhuang, China; 5 Department of Cardiology, The First Hospital of Hebei Medical University, Shijiazhuang, Hebei, China; 6 Department of Integrative Biology and Pharmacology, University of Texas Health Science Center at Houston, Houston, Texas, USA

**Keywords:** ORP2, atherosclerosis, macrophages, foam cell formation, cholesterol efflux, LXRα

## Abstract

Cholesterol-loaded macrophage foam cells are a key feature of atherosclerotic plaques. Oxysterol-binding protein-related protein 2 (ORP2) facilitates the transport of cholesterol from lysosomes to the plasma membrane in cultured cell lines. However, the role of ORP2 in macrophages and its involvement in atherosclerosis remain unclear. In this study, we found ORP2 expression was reduced in atherosclerotic vessels and in macrophages exposed to oxidized LDL (ox-LDL). Myeloid-specific human ORP2 overexpression (hORP2^MOE^) mice were generated and crossed with atherosclerotic-prone ApoE^−/−^ mice and then fed a high-fat diet (HFD) to induce atherosclerosis. Our results showed that myeloid-specific hORP2 overexpression significantly reduced the atherosclerotic plaque area, along with reduced lipid accumulation, necrotic core size, birefringent crystals, and macrophage presence within the plaque. Additionally, hORP2 overexpression in peritoneal macrophages (PMCs) led to reduced lipid accumulation and increased expression of key cholesterol efflux proteins, including LXRα, ABCA1, and ABCG1. Furthermore, hOPR2 overexpression promoted NBD-cholesterol efflux from macrophages. To explore the underlying mechanism, we conducted co-immunoprecipitation, immunofluorescence, and cytoplasmic/nuclear fractionation experiments. Our findings revealed that ORP2 interacts with LXRα and promotes its nuclear localization in macrophages. Moreover, the LXR antagonist GSK2033 blocked the reduction in foam cell formation and the increase in LXRα nuclear translocation induced by hORP2 overexpression. These findings suggest that ORP2 interacts with LXRα and facilitates its nuclear translocation in macrophages, leading to reduced foam cell formation and alleviation of atherosclerosis.

Atherosclerosis, a chronic inflammatory disease, is a leading cause of cardiovascular and cerebrovascular diseases, representing a major threat to human health. Macrophages, key immune cells in the arterial wall, play a central role in the initiation and progression of atherosclerosis (1). In the early stages of the disease, macrophages internalize modified lipoproteins, particularly oxidized low-density lipoproteins (ox-LDL), leading to the formation of lipid-laden foam cells, a hallmark of atherosclerotic lesions. These foam cells are present throughout all stages of lesion development. In contrast, macrophage cholesterol efflux and reverse cholesterol transport mediated by high-density lipoprotein (HDL) can mitigate foam cell formation, thereby reducing the occurrence and progression of atherosclerosis (1, 2). Additionally, the phenotypic plasticity between pro-inflammatory M1 macrophages and anti-inflammatory M2 macrophages plays a crucial role in regulating macrophage function and inflammatory status, influencing the course of atherosclerosis (3). Liver X receptors (LXRs), activated by oxysterols, are key regulators of cholesterol homeostasis in macrophages. LXR activation promotes the expression of cholesterol efflux-related genes, such as ABCA1 and ABCG1, thereby inhibiting foam cell formation and improving atherosclerotic progression. Beyond their role in cholesterol transport, LXRs also exert anti-inflammatory effects that contribute to the attenuation of foam cell formation and atherosclerosis (4, 5).

The oxysterol-binding protein (OSBP) and its related proteins (ORPs) are a family of proteins that play essential roles in regulating cholesterol transport within cells. This protein family, consisting of 12 members in humans (OSBP and ORP1-11), includes a conserved oxysterol-binding domain (ORD) at the C-terminus, which enables the binding of oxysterols. OSBP/ORPs are evolutionarily conserved and participate in diverse cellular processes such as lipid metabolism, vesicle trafficking, growth regulation, and signal transduction (6, 7). Several ORPs, including ORP1L, ORP4L, and ORP8, have been implicated in regulating macrophage cholesterol homeostasis and atherosclerosis. However, the roles of these proteins in atherosclerosis are complex and, in some cases, contradictory. For instance, while ORP1L overexpression has been associated with reduced cholesterol efflux and exacerbated atherosclerosis in atherosclerosis-prone mouse models (8), suppression of ORP1L in macrophages has been shown to impair cholesterol efflux (9). ORP8 can inhibit the expression of ABCA1 in macrophages, leading to a reduction in cholesterol efflux (10), and bone marrow cell-specific ORP8 knockout can decrease the progression of atherosclerosis (11). Additionally, ORP4L maintains the expression of anti-apoptotic Bcl-XL in macrophages, and ORP4L knockout mice exhibited increased macrophage apoptosis and reduced atherosclerotic lesions (12).

ORP2, a 55-kDa protein encoded by *Osbpl2*, is a member of the ORP subfamily II and is expressed in various tissues, including macrophages. Unlike other mammalian ORPs, ORP2 lacks a membrane-targeting pleckstrin homology (PH) domain and a transmembrane domain. ORP2 binds cholesterol, oxysterols, and phosphatidylinositol, regulating the transport of sterols between various cellular compartments, including the plasma membrane, late endosomes/lysosomes (LE/Lys), the endoplasmic reticulum, and lipid droplets (13, 14, 15). Previous work has shown that ORP2 plays a role in delivering cholesterol from lysosomes to the plasma membrane (13). In HuH7 hepatocytes, ORP2 interacts with the cytoskeletal component F-actin, promoting cell adhesion, migration, and proliferation. Furthermore, ORP2 forms a complex with key regulators of AKT signaling, and its deletion results in the suppression of AKT activity, leading to impaired glucose uptake and metabolism, as well as reduced triglyceride synthesis and storage (16, 17). In addition to its role in cellular signaling, ORP2 has been implicated in various aspects of cholesterol metabolism. For example, overexpression of ORP2 in HeLa and CHO cells leads to increased cholesterol efflux, decreased cholesterol esterification, and reduced ACAT activity. Furthermore, ORP2 enhances the expression of LDL receptors, promoting cholesterol uptake (18, 19). ORP2 has also been shown to regulate cholesterol synthesis *via* the AMPK/SP1/SREBP2 pathway (20). Studies conducted in H295R adrenal cortex cell lines have demonstrated that ORP2 interacts with the liver X receptor (LXR), facilitating its nuclear translocation and thereby activating the transcription of downstream target genes, such as ABCA1 (21).

Despite these insights, existing studies on ORP2 have been conducted exclusively in immortalized or non-macrophage cell lines under non-physiological conditions. Whether ORP2 plays a comparable role in primary macrophages—the primary mediators of foam cell formation and atherogenesis—remains unknown. In particular, the physiological relevance of ORP2-LXR signaling *in vivo* within the context of macrophage-driven cholesterol efflux has not been explored.

In this study, we investigate the role of ORP2 in macrophage lipid metabolism and atherosclerosis using myeloid-specific ORP2 overexpression model. By employing LysM-Cre-driven expression of human ORP2 in macrophages and combining this with ApoE^−/−^ atherosclerosis-prone mice, we reveal that ORP2 enhances the nuclear translocation of LXRα in macrophages, promoting cholesterol efflux and reducing foam cell formation. This represents the first *in vivo* demonstration of ORP2 function in primary macrophages, offering mechanistic insights into its role in cholesterol homeostasis and atheroprotection. Our findings suggest that ORP2 plays a protective role in the pathogenesis of atherosclerosis by modulating cholesterol homeostasis in macrophages.

## Results

### Decreased ORP2 expression in atherosclerotic vessels and ox-LDL-exposed macrophages

**Figure 1:**
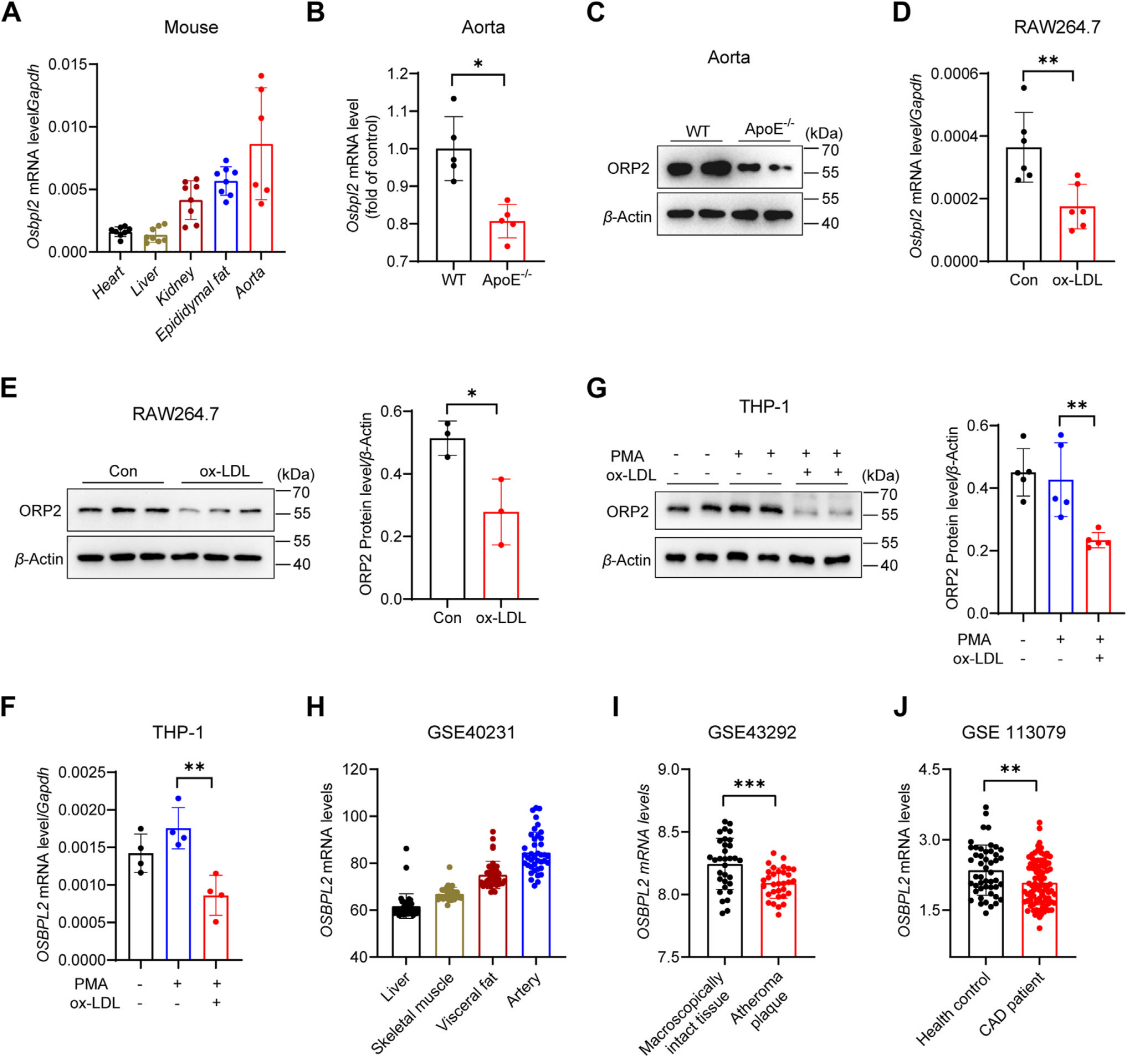
**Decreased ORP2 expression in atherosclerotic aorta and ox-LDL exposed macrophages.***A*, the *Osbpl2* mRNA expression levels in different tissues of C57BL/6J mice (n = 6–8). *B*, the mRNA expression levels of *Osbpl2* in aorta of 32 to 34 weeks old WT and ApoE^−/−^ mice on C57BL/6J background (n = 5). (Data were obtained from GEO database: http://www.ncbi.nlm.nih.gov/geo/, Data set number was GSE2372 and GSE19286). Data are presented as mean ± SD. Unpaired Student's *t* test was used. ∗*p* < 0.05. *C*, the protein expression of ORP2 in the aorta of 28 weeks old WT and ApoE^−/−^ mice (n = 2). *D*, the mRNA expression level of *Osbpl2* in RAW264.7 cells incubation with 50 μg/ml ox-LDL for 24 h (n = 6). Data are presented as mean ± SD. Unpaired Student's *t* test was used. ∗∗*p* < 0.01. *E*, the protein expression level of ORP2 in RAW264.7 cells incubation with 50 μg/ml ox-LDL for 24 h (n = 3). Data are presented as mean ± SD. Unpaired Student's *t* test was used. ∗*p* < 0.05. *F*, the mRNA expression level of *Osbpl2* in THP-1 cells (activated into macrophages by PMA, 75 ng/ml for 72 h) incubation with 50 μg/ml ox-LDL for 24 h (n = 4). Data are presented as mean ± SD. Statistical significance was determined by one-way ANOVA with Tukey's multiple comparison test. ∗∗*p* < 0.01. *G*, the protein expression level of ORP2 in THP-1 (activated into macrophages by PMA, 75 ng/ml for 72 h) incubation with 50 μg/ml ox-LDL for 24 h (n = 5). Data are presented as mean ± SD. Statistical significance was determined by one-way ANOVA with Tukey's multiple comparison test. ∗∗*p* < 0.01. *H*, analysis of GEO database (GSE40231) for *OSBPL2* mRNA expression in human liver, skeletal muscle, visceral fat, and Internal mammary artery (n = 40). *I*, analysis of GEO database (GSE43292) for *OSBPL2* mRNA expression in human atheroma plaque and macroscopically intact tissue (n = 32). Data are presented as mean ± SD. Unpaired Student's *t* test was used. ∗∗∗*p* < 0.001. *J*, analysis of GEO database (GSE113079) for *OSBPL2* mRNA expression in peripheral blood mononuclear cells collected from healthy individuals or patients with carotid atherosclerotic disease (n = 48–93). Data are presented as mean ± SD. Unpaired Student's *t* test was used. ∗∗*p* < 0.01.

To investigate the role of ORP2 in atherosclerosis, we first examined the mRNA expression of *Osbpl2*, the gene encoding the ORP2 protein, in various mouse tissues. Our results showed that *Osbpl2* mRNA expression was higher in the aorta of C57BL/6J mice compared to the heart, liver, kidney, and epididymal fat (Fig. 1*A*). Next, we assessed ORP2 expression in the aorta of atherosclerotic mice. Both mRNA and protein levels of ORP2 were reduced in the aorta of ApoE knockout (ApoE^−/−^) mice with atherosclerotic lesions compared to wild-type (WT) mice without lesions (Fig. 1, *B* and *C*). To further investigate the effects of atherosclerotic stimuli on ORP2 expression in macrophages, we utilized the RAW264.7 murine macrophage cell line and the PMA-activated THP1 human monocyte cell line. Our results demonstrated that incubation with oxidized LDL (ox-LDL) for 24 h led to a significant reduction in both ORP2 mRNA and protein levels in RAW264.7 cells (Fig. 1, *D* and *E*), as well as in PMA-activated THP1 cells (Fig. 1, *F* and *G*). Finally, we analyzed human *OSBPL2* expression in several public databases. The microarray results from the GSE40231 dataset revealed that *OSBPL2* mRNA expression was higher in the artery compared to the liver, skeletal muscle, and visceral fat (Fig. 1*H*). Furthermore, the GSE43292 dataset showed a decrease in *OSBPL2* expression in atheroma plaques compared to control tissues (Fig. 1*I*). Additionally, *OSBPL2* expression in peripheral blood mononuclear cells was reduced in patients with coronary artery disease as indicated by the GSE113079 dataset (Fig. 1*J*). These findings suggest that ORP2 expression is downregulated in macrophages exposed to atherosclerotic stimuli, potentially implicating ORP2 in foam cell formation and atherosclerosis.

### Generation of myeloid-specific human ORP2 overexpression mice

**Figure 2:**
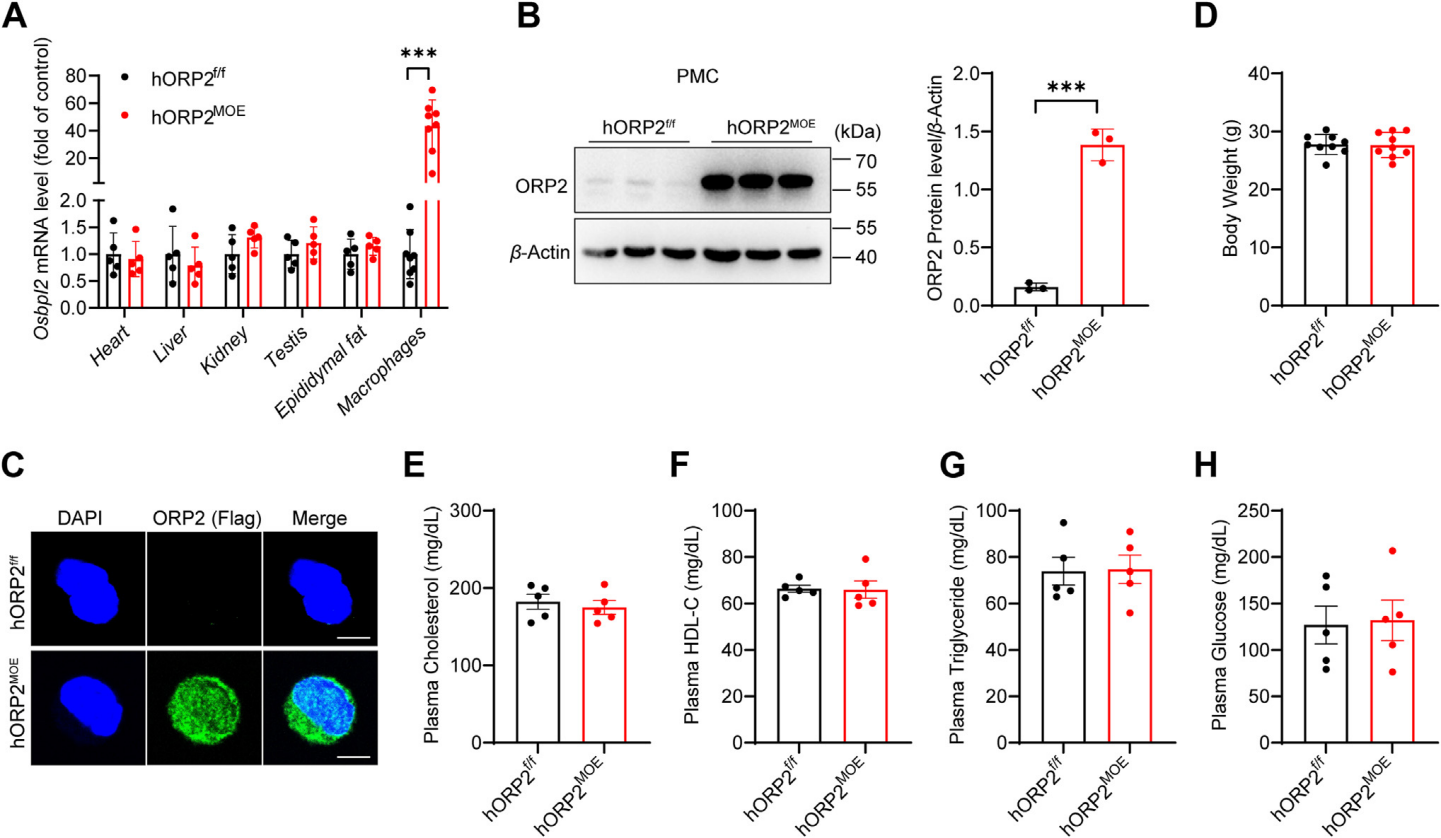
**Characterization of myeloid-specific human ORP2 overexpression mice.***A*, the mRNA expression levels of *Osbpl2* in different tissues from hORP2^f/f^ and hORP2^MOE^ mice (n = 5–8 per group). Data are presented as mean ± SD. Unpaired Student's *t* test was used. ∗∗∗*p* < 0.001. *B*, the protein expression of ORP2 in peritoneal macrophages from hORP2^f/f^ and hORP2^MOE^ mice (n = 3). Data are presented as mean ± SD. Unpaired Student's *t* test was used. ∗∗∗*p* < 0.001. *C*, representative immunofluorescence staining for ORP2-Flag (green) in peritoneal macrophages from hORP2^f/f^ and hORP2^MOE^ mice. Scale bar = 5 μm. *D*, body weight of 8-week-old male hORP2^f/f^ and hORP2^MOE^ mice (n = 9). *E–H*, plasma total cholesterol (*E*), HDL-C (*F*), triglyceride (*G*), and glucose (*H*) levels of 8 weeks old male hORP2^f/f^ and hORP2^MOE^ mice (n = 5).

To investigate the role of ORP2 in macrophages, we first generated human ORP2 knock-in mice by introducing human ORP2 downstream of a stop cassette flanked by loxP sites into the murine Rosa26 locus through gene targeting, resulting in hORP2 floxed (hORP2^f/f^) mice. These hORP2^f/f^ mice were then crossed with LysM^cre^ transgenic mice to create myeloid-specific human ORP2 overexpression (hORP2^MOE^) mice (Fig. S1). To verify ORP2 overexpression in macrophages, we assessed the mRNA levels of Osbpl2 (using primers that recognize both human and mouse Osbpl2) in various tissues including the heart, liver, kidney, testis, epididymal fat, and peritoneal macrophages (PMCs) of both hORP2^f/f^ and hORP2^MOE^ mice. We observed a significant increase in Osbpl2 mRNA expression in the macrophages of hORP2^MOE^ mice, while expression in other tissues remained unaffected (Fig. 2*A*). Western blot analysis further confirmed a marked elevation in ORP2 protein levels in the PMCs of hORP2^MOE^ mice (Fig. 2*B*). To further validate this, immunofluorescence staining revealed increased ORP2 expression in the PMCs of hORP2^MOE^ mice, with the protein distributed in both the cytoplasm and nucleus (Fig. 2*C*).

Since previous studies have shown that ORP2 regulates cholesterol homeostasis as well as triglyceride and carbohydrate metabolism (16), we assessed the body weight, blood lipid, and glucose levels in 8-week-old male hORP2^MOE^ mice fed a normal chow diet. We found no significant differences in body weight, plasma total cholesterol, high-density lipoprotein cholesterol (HDL-C), triglyceride, or blood glucose levels between hORP2^MOE^ mice and controls (Fig. 2, *D*–*H*). These findings indicate that we have successfully established myeloid-specific hORP2 overexpression mice, which maintain normal blood lipid and glucose levels.

### ORP2 overexpression in macrophages prevents diet-induced atherosclerosis

**Figure 3:**
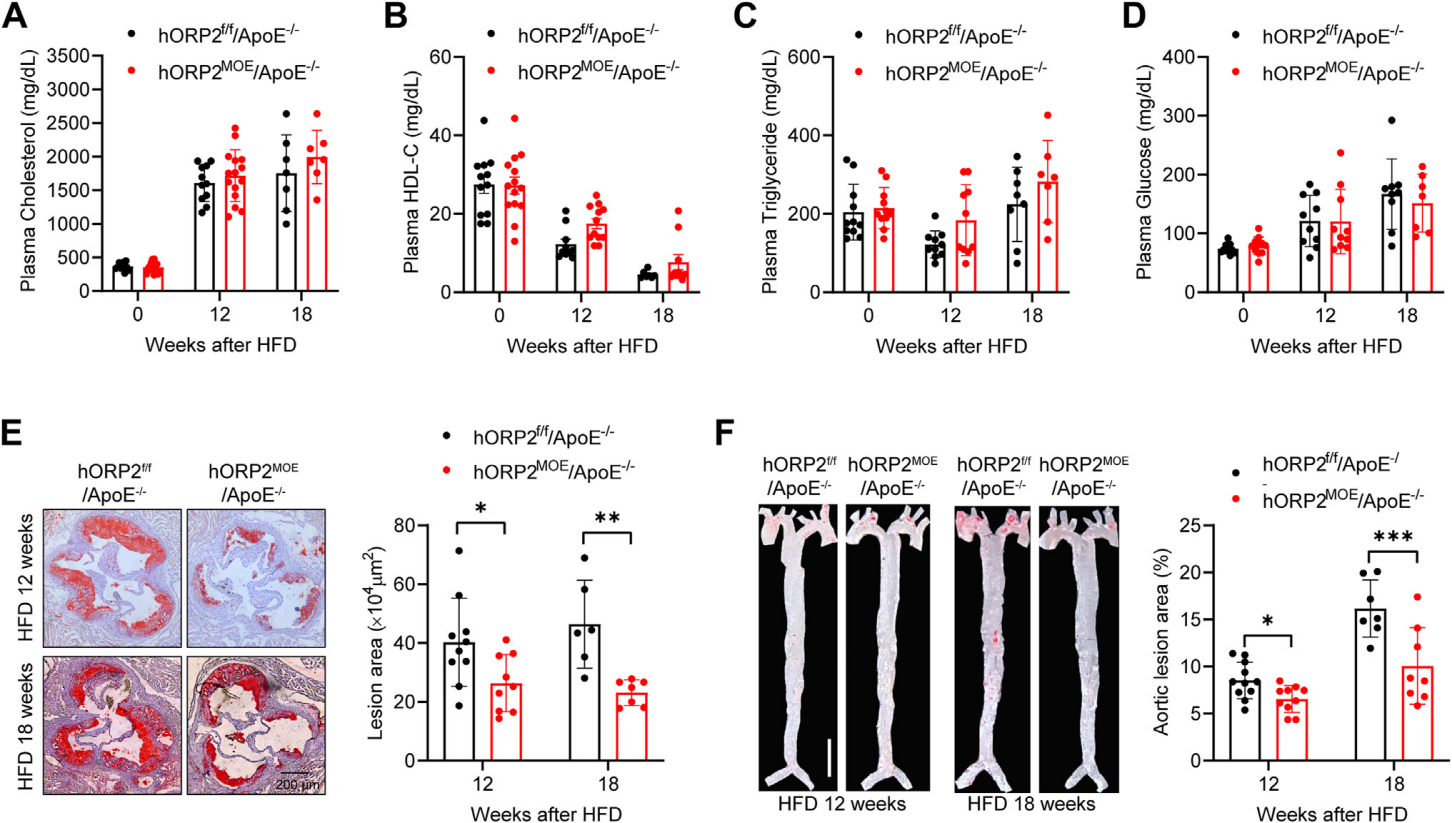
**Improved atherosclerotic lesions in myeloid-specific hORP2 overexpression mice.***A–D*, plasma total cholesterol (*A*), HDL-C (*B*), triglyceride (*C*), and glucose (*D*) levels of male hORP2^f/f^/ApoE^−/−^ and hORP2^MOE^/ApoE^−/−^ mice fed with Western-type high-fat diet (HFD) for 12 or 18 weeks (n = 7–15 per group). *E*, representative Oil-Red-O staining of aortic root sections from male hORP2^f/f^/ApoE^−/−^ and hORP2^MOE^/ApoE^−/−^ mice fed with HFD for 12 or 18 weeks and quantification of atherosclerotic plaque areas (n = 6–10 per group). Scale bar = 200 μm. Data are presented as mean ± SD. Statistical significance was determined by two-way ANOVA with Sidak's multiple comparison test. ∗*p* < 0.05, ∗∗*p* < 0.01. *F*, representative Oil-Red O-stained *En face* images of whole aorta from male hORP2^f/f^/ApoE^−/−^ and hORP2^MOE^/ApoE^−/−^ mice fed with HFD for 12 or 18 weeks. Scale bar = 0.5 cm. Lesion area was quantified as percentage of total surface area of aorta (n = 7–11 per group). Data are presented as mean ± SD. Statistical significance was determined by two-way ANOVA with Sidak's multiple comparison test. ∗*p* < 0.05, ∗∗∗*p* < 0.001.

To investigate the effect of macrophage-specific ORP2 overexpression on hyperlipidemia and atherosclerosis, we crossed hORP2^MOE^ mice with ApoE^−/−^ mice, which are prone to hypercholesterolemia and atherosclerosis, to generate hORP2^MOE^/ApoE^−/−^ mice. Male mice were then placed on a Western-type high-fat diet (HFD) for 12 or 18 weeks to induce hyperlipidemia and atherosclerosis. Plasma lipid and glucose levels were measured, and no significant differences in total cholesterol, HDL-C, triglycerides, or glucose were observed between hORP2^MOE^/ApoE^−/−^ and hORP2^f/f^/ApoE^−/−^ control mice (Fig. 3, *A*–*D*). To further assess the role of macrophage ORP2 in atherosclerosis, we analyzed atherosclerotic lesions in hORP2^MOE^/ApoE^−/−^ and control mice after 12 or 18 weeks of HFD feeding. Oil Red O staining revealed that atherosclerotic plaques in the aortic root and whole aorta of hORP2^MOE^/ApoE^−/−^ mice were significantly smaller compared to those in hORP2^f/f^/ApoE^−/−^ control mice (Fig. 3, *E* and *F*).

**Figure 4:**
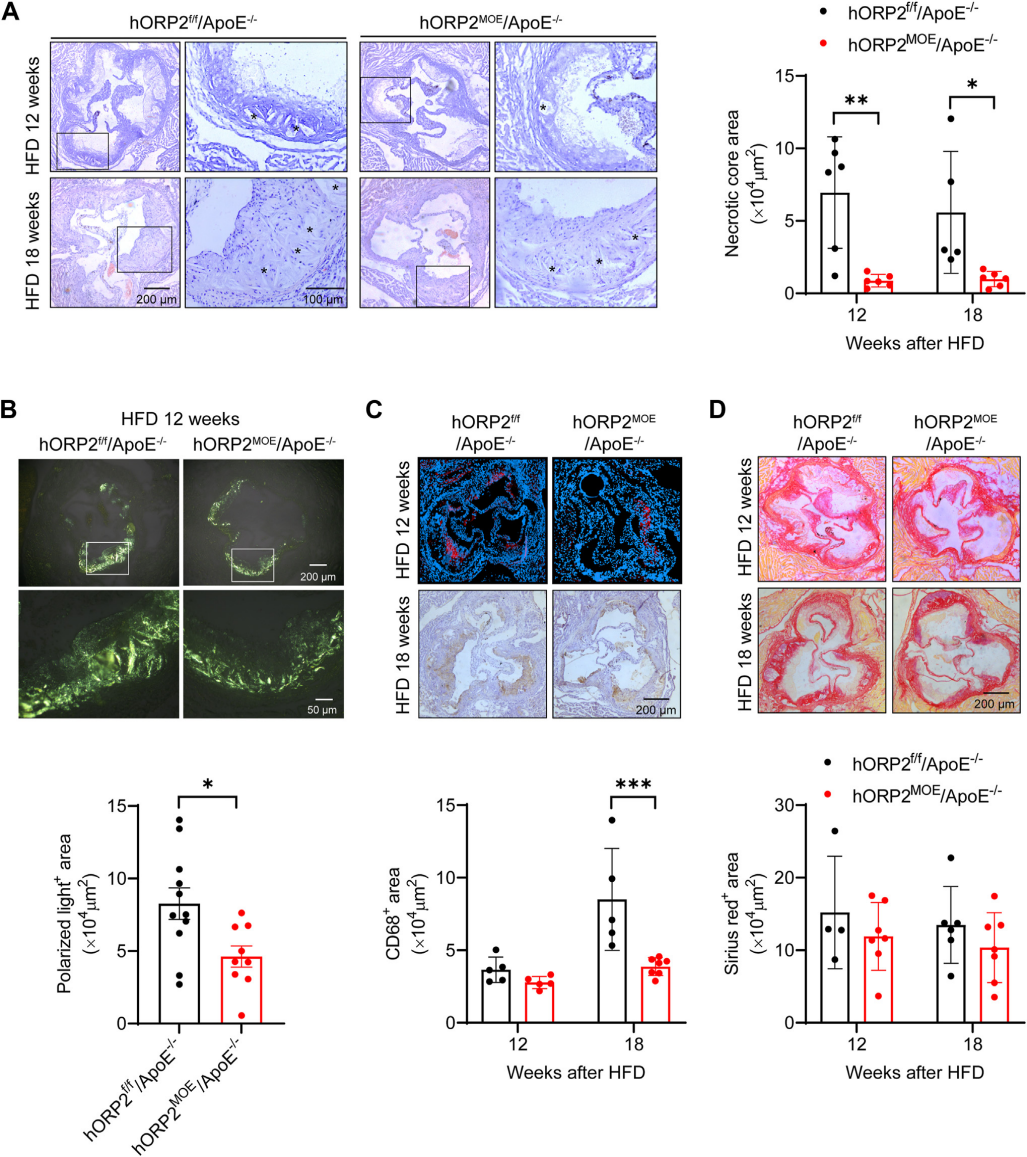
**Reduced necrotic core and cholesterol crystals in atherosclerotic lesions of myeloid-specific hORP2 overexpression mice.***A*, representative H&E staining of aortic root sections from male hORP2^f/f^/ApoE^−/−^ and hORP2^MOE^/ApoE^−/−^ mice fed with HFD for 12 or 18 weeks and quantification of necrotic core area in atherosclerotic plaque (n = 5–6 per group). Scale bar = 200 μm (*left*), 100 μm (*right*). Data are presented as mean ± SD. Statistical significance was determined by two-way ANOVA with Sidak's multiple comparison test. ∗*p* < 0.5, ∗∗*p* < 0.1. *B*, representative polarization microscopy images of aortic root sections from male hORP2^f/f^/ApoE^−/−^ and hORP2^MOE^/ApoE^−/−^ mice fed with HFD for 12 weeks and quantification of polarized light-positive areas in atherosclerotic plaque (n = 9–11 per group). Scale bar = 200 μm (*top*), 50 μm (*bottom*). Data are presented as mean ± SD. Unpaired Student's *t* test was used. ∗*p* < 0.05. *C*, representative CD68 immunohistochemical or immunofluorescence staining of aortic root sections from male hORP2^f/f^/ApoE^−/−^ and hORP2^MOE^/ApoE^−/−^ mice fed with HFD for 12 or 18 weeks and quantification of lesion macrophage content. Scale bar = 200 μm. (n = 5–7 per group). *D*, representative Sirius red staining of aortic root sections from male hORP2^f/f^/ApoE^−/−^ and hORP2^MOE^/ApoE^−/−^ mice fed with HFD for 12 or 18 weeks and quantification of lesion collagen content. Scale bar = 200 μm. (n = 5–7 per group).

To evaluate the impact of ORP2 on the composition and stability of atherosclerotic plaques, we performed H&E staining on aortic root sections and quantified the necrotic core area. The results showed that hORP2^MOE^/ApoE^−/−^ mice had a significantly smaller necrotic core in their atherosclerotic lesions (Fig. 4*A*). Additionally, polarization microscopy revealed fewer birefringent crystals (suggestive of cholesterol crystals) in the plaques of hORP2^MOE^/ApoE^−/−^ mice (Fig. 4*B*). Furthermore, we examined macrophage infiltration and collagen content in the atherosclerotic lesions at the aortic root. We found a significant reduction of CD68^+^ macrophages in the plaques of hORP2^MOE^/ApoE^−/−^ mice after 18 weeks on an HFD (Fig. 4*C*). However, there was no significant difference in Sirius Red staining for collagen content between plaques of hORP2^MOE^/ApoE^−/−^ and hORP2^f/f^/ApoE^−/−^ mice (4D). These findings suggest that overexpression of hORP2 specifically in macrophages can attenuate high-fat diet-induced atherosclerosis and enhance the stability of atherosclerotic plaques.

### ORP2 overexpression in macrophages promotes cholesterol efflux

**Figure 5:**
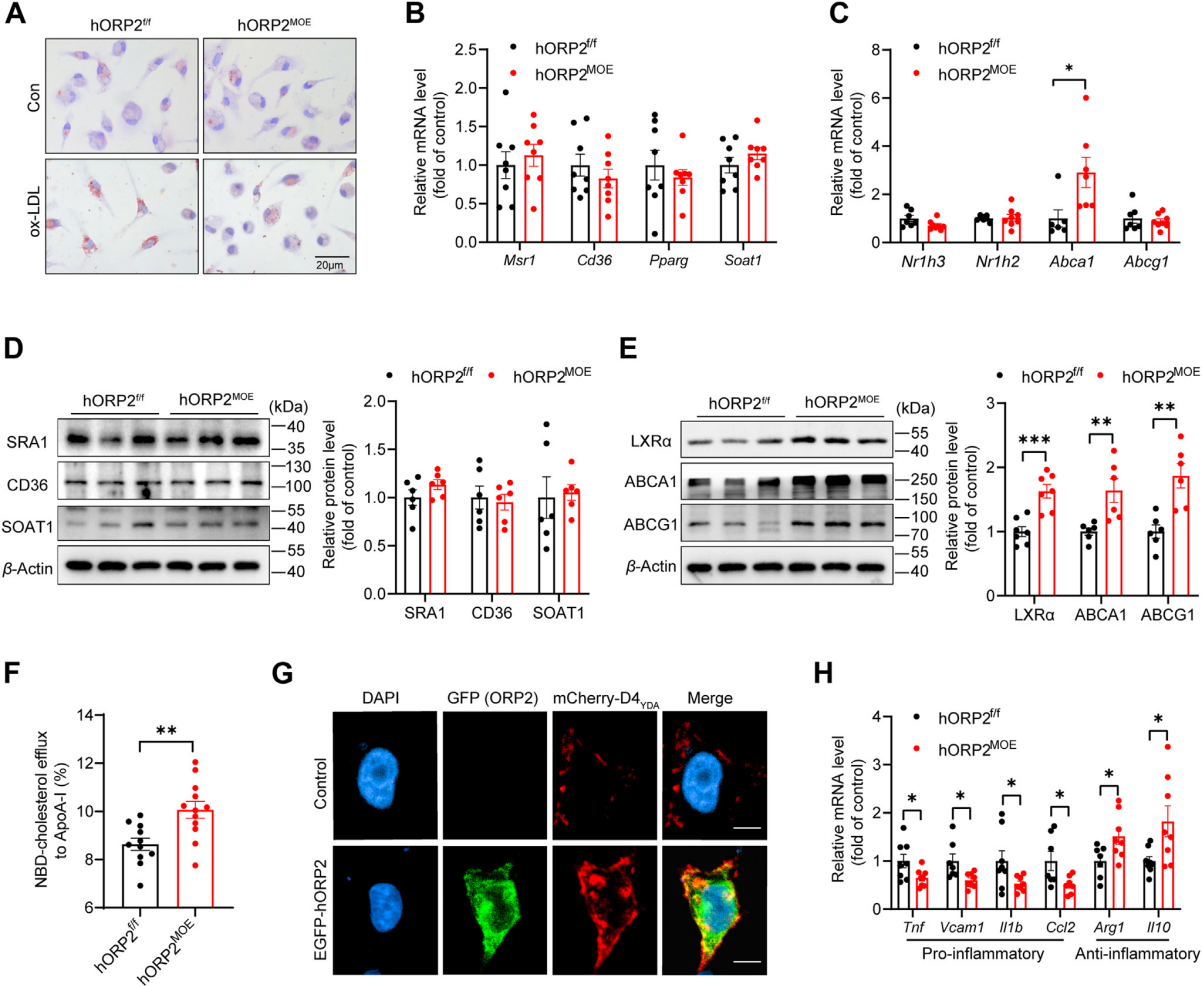
**ORP2 overexpression in macrophages promotes cholesterol efflux.***A*, representative images of Oil Red O staining of peritoneal macrophages from hORP2^f/f^ and hORP2^MOE^ mice with or without ox-LDL (25 μg/ml) incubation for 24 h. Scale bar = 20 μm. *B* and *C*, relative mRNA expression levels of genes regulating cholesterol uptake and esterification (*B*), and efflux (*C*) in peritoneal macrophages from hORP2^f/f^ and hORP2^MOE^ mice incubated with ox-LDL (25 μg/ml) for 24 h (n = 6–8). Data are presented as mean ± SD. Unpaired Student's *t* test was used. ∗*p* < 0.05. *D–E*, relative expression levels of proteins related to cholesterol uptake and esterification (*D*), and efflux (*E*) in peritoneal macrophages from hORP2^f/f^ and hORP2^MOE^ mice incubated with ox-LDL (25 μg/ml) for 24 h (n = 6–7). Data are presented as mean ± SD. Unpaired Student's *t* test was used. ∗∗*p* < 0.1, ∗∗∗*p* < 0.01. *F*, ApoA-I mediated cholesterol efflux. Peritoneal macrophages from hORP2^f/f^ and hORP2^MOE^ mice were labeled with NBD-cholesterol (5 μM) overnight, followed by 4 or 6 h efflux to ApoA-I (10 μg/ml). The efflux rate (%) indicates the fluorescence signal (FI) in the medium divided by the total FI in medium and cells (n = 11–12). Data are presented as mean ± SD. Unpaired Student's *t* test was used. ∗∗*p* < 0.01. *G*, representative image of HEK293T cells overexpressing ORP2-EGFP. mCherry-D4_YDA_ was used as a biosensor of plasma membrane cholesterol (n = 3 independent experiments). Scale bar = 10 μm. *H*, relative mRNA expression levels of pro-inflammatory and anti-inflammatory genes in peritoneal macrophages from hORP2^f/f^ and hORP2^MOE^ mice incubated with ox-LDL (25 μg/ml) for 24 h (n = 7–eight per group). Data are presented as mean ± SD. Unpaired Student's *t* test was used. ∗*p* < 0.05.

Macrophage uptake of modified lipoproteins is a crucial process in the development and progression of atherosclerosis. In this study, we incubated PMCs with ox-LDL and assessed foam cell formation using Oil-Red-O staining. Our findings revealed a significant reduction in positive staining in hORP2^MOE^ cells compared to the control group (Fig. 5*A* and Fig. S2*A*), suggesting that ORP2 inhibits ox-LDL-induced foam cell formation in macrophages.

Foam cell formation is influenced by three key processes: cholesterol uptake, esterification, and efflux. To explore these pathways, we analyzed the expression of key genes in ox-LDL-incubated PMCs. The results showed no significant differences in the mRNA levels of genes associated with cholesterol uptake and esterification, such as *Msr1*, *Cd36*, *Pparg,* and *Soat1*, between hORP2^MOE^ and control (hORP2^f/f^) cells (Fig. 5*B*). However, the mRNA expression of *Abca1*, a critical gene involved in cholesterol efflux, was significantly upregulated in hORP2^MOE^ cells (Fig. 5*C*). Additionally, protein levels of cholesterol uptake and esterification markers (SRA1, CD36, and SOAT1) were comparable between hORP2^MOE^ cells and the control group (Fig. 5*D*). In contrast, proteins related to cholesterol efflux, including LXRα, ABCA1, and ABCG1, were significantly elevated in hORP2^MOE^ cells (Fig. 5*E*).

To further investigate ORP2’s role in macrophage cholesterol efflux, we conducted an NBD-cholesterol efflux assay using ApoA-I as the acceptor. The results showed that the cholesterol efflux rate was significantly higher in hORP2^MOE^ cells compared to control cells (Fig. 5*F*). Given that ORP2 has been previously shown to facilitate cholesterol transport to the plasma membrane, we overexpressed hORP2 in HEK293T cells and labeled plasma membrane cholesterol with D4_YDA_. Overexpression of hORP2 led to increased cholesterol levels on the plasma membrane, which could contribute to enhanced cholesterol efflux (Fig. 5*G*). Taken together, these findings suggest that ORP2 inhibits foam cell formation by enhancing macrophage cholesterol efflux.

In addition to cholesterol efflux, we explored the impact of ORP2 on macrophage phenotypic switching, particularly the balance between pro-inflammatory and anti-inflammatory responses. After incubating PMCs with ox-LDL, we assessed the expression of inflammatory genes. Our results revealed a significant reduction in the expression of pro-inflammatory genes such as *Tnf*, *Vcam1*, *Il1b*, and *Ccl2* in hORP2^MOE^ cells, while anti-inflammatory markers (*Arg1* and *Il10*) were significantly upregulated (Fig. 5*H*). These findings suggest that macrophages overexpressing ORP2 adopt an anti-inflammatory phenotype, which may contribute to limiting the progression of atherosclerosis.

### ORP2 overexpression prevents macrophage foam cell formation *via* LXR**α**

**Figure 6:**
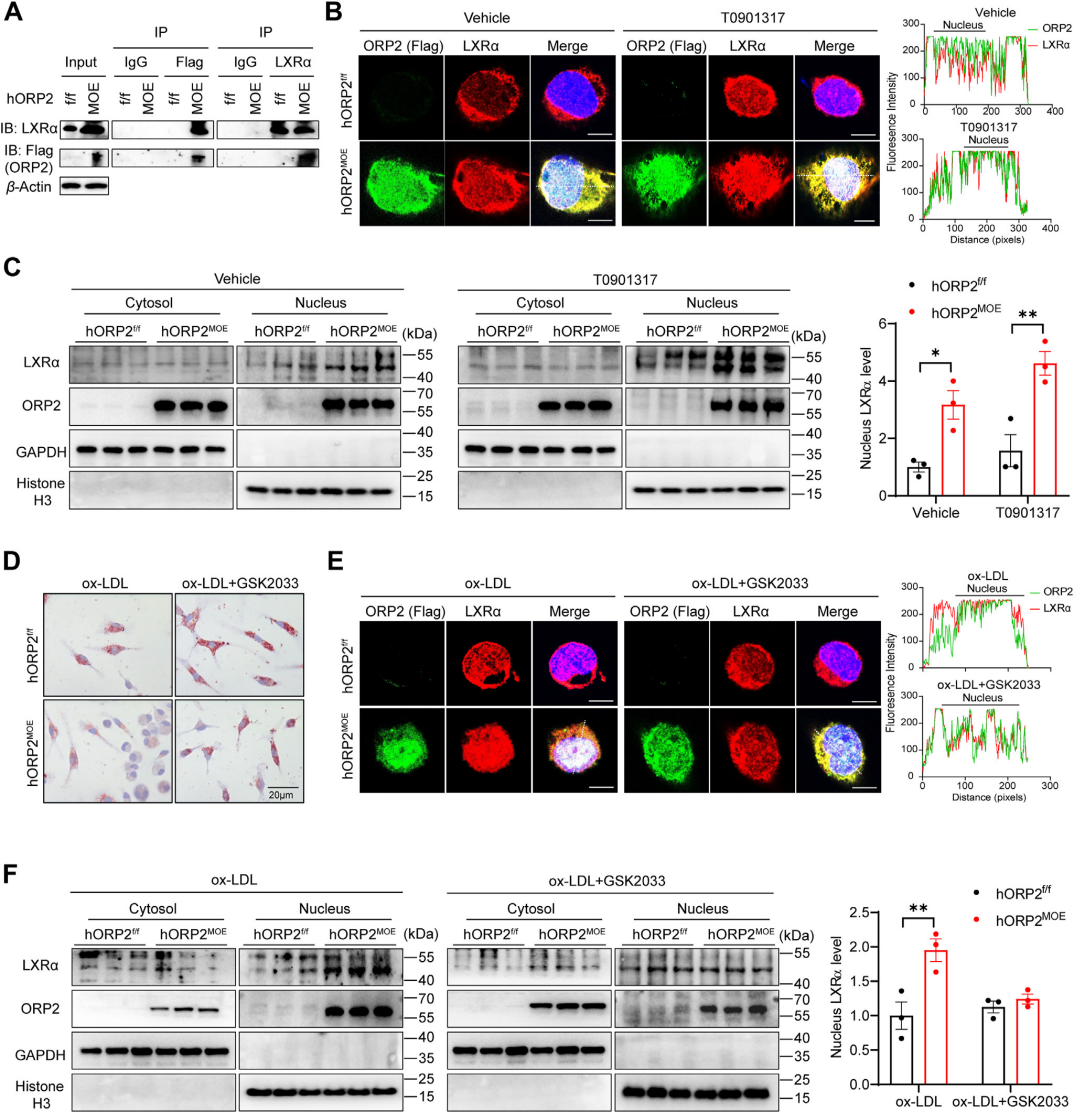
**ORP2 interacts with LXRα and facilitates its nuclear translocation.***A*, CO-IP of ORP2 (Flag) and LXRα in peritoneal macrophages from hORP2^f/f^ and hORP2^MOE^ mice. *B*, representative immunofluorescence staining for ORP2-Flag (*green*) and LXRα (*red*) in peritoneal macrophages from hORP2^f/f^ and hORP2^MOE^ mice treated with or without T0901317 (5 μM) for 24 h (n = 3), Scale bar = 5 μm. Shown on the right are line profiles of fluorescence intensities of ORP2 and LXRα across the white dashed lines. *C*, Western blot for the expression of LXRα and ORP2 in cytoplasmic and nucleus fractions of peritoneal macrophages from hORP2^f/f^ and hORP2^MOE^ mice treated with or without T0901317 (5 μM) for 24 h (n = 3). Data are presented as mean ± SD. Statistical significance was determined by two-way ANOVA with Tukey's multiple comparison test. ∗*p* < 0.5. *D–F*, peritoneal macrophages from hORP2^f/f^ and hORP2^MOE^ mice were treated with or without LXR antagonist GSK2033 (30 μg/ml) for 24 h, then incubated with ox-LDL (25 μg/ml) for 24 h. *D*, representative images of Oil-Red-O staining. Scale bar = 20 μm. *E*, representative immunofluorescence staining for ORP2-Flag (*green*) and LXRα (*red*), Scale bar = 5 μm. Shown on the right are line profiles of fluorescence intensities of ORP2 and LXRα across the white dashed lines. *F*, LXRα protein level of primary peritoneal macrophages in cytosol and nuclear respectively (n = 3). Data are presented as mean ± SD. Statistical significance was determined by two-way ANOVA with Sidak's multiple comparison test. ∗*p* < 0.05, ∗∗*p* < 0.01.

LXRα, a key nuclear receptor that regulates cholesterol efflux, plays a crucial role in macrophage foam cell formation. To investigate whether LXRα mediates the effects of ORP2 on foam cell formation, we performed immunoprecipitation experiments to examine the interaction between ORP2 (Flag) and LXRα in PMCs from hORP2^MOE^ mice (Fig. 6*A*). Additionally, we used immunofluorescence staining to confirm this interaction and observed that ORP2 overexpression promoted the nuclear translocation of LXRα. Treatment with the LXR agonist T0901317 further enhanced the nuclear localization of both LXRα and ORP2 in hORP2^MOE^ macrophages (Fig. 6*B*).

To further validate the impact of ORP2 on LXRα localization, we conducted Western blot analysis to measure the protein levels of LXRα in the cytoplasmic and nuclear fractions of PMCs. While there was no difference in the cytoplasmic levels of LXRα between hORP2^MOE^ and control cells, the nuclear content of LXRα was significantly higher in hORP2^MOE^ cells compared to hORP2^f/f^ cells (Fig. 6*C*). These findings suggest that ORP2 interacts with LXRα and facilitates its nuclear translocation.

To further explore how ORP2 overexpression affects macrophages through LXR activation, we pretreated PMCs with an LXR antagonist, GSK2033, for 24 h prior to ox-LDL stimulation. Oil-Red-O staining results showed that the positive staining in hORP2^MOE^ PMCs pretreated with GSK2033 was comparable to that in hORP2^f/f^ cells (Figs 6*D* and S2*B*), indicating that the LXR antagonist prevented the inhibitory effect of ORP2 overexpression on foam cell formation. Immunofluorescence staining also revealed that the nuclear localization of LXRα was not enhanced in hORP2^MOE^ PMCs pretreated with GSK2033, compared to control cells (Fig. 6*E*). Western blot analysis of LXRα protein levels in the cytoplasmic and nuclear fractions of PMCs confirmed these results, showing that the LXR antagonist blocked the effect of ORP2 overexpression on LXRα nuclear translocation (Fig. 6*F*).

In conclusion, these results suggest that ORP2 prevents foam cell formation by promoting the nuclear translocation of LXRα, highlighting the importance of ORP2-LXRα signaling in regulating macrophage lipid metabolism.

## Discussion

ORP2, a member of the oxysterol-binding protein family, plays a crucial role in cholesterol metabolism. However, its specific involvement in macrophage cholesterol homeostasis and atherosclerosis has not been well explored. To our knowledge, this study is the first to investigate the functional role of ORP2 in primary macrophages and within the *in vivo* context of atherosclerosis, thereby extending previous findings from non-macrophage cell lines into a disease-relevant physiological setting.

In this study, we investigated the effects of ORP2 in macrophages using myeloid-specific ORP2 overexpression mice and primary peritoneal macrophage cells (PMCs). Our findings demonstrate that ORP2 overexpression enhances cholesterol efflux from macrophages, inhibits foam cell formation, and impedes the progression of atherosclerosis. Further investigations revealed that ORP2 interacts with LXRα in macrophages, promoting its nuclear translocation and effectively inhibiting foam cell formation.

To explore the role of ORP2 in atherosclerosis, we generated myeloid-specific human ORP2 overexpression mice. These mice were then crossbred with atherosclerotic-prone ApoE^−/−^ mice and fed a Western-type high-fat diet (HFD) to induce atherosclerosis. Our results indicate that myeloid-specific hORP2 overexpression significantly reduces the atherosclerotic lesion area in ApoE^−/−^ mice, suggesting a protective effect of ORP2 against atherosclerosis. In advanced atherosclerosis, foam macrophages are more prone to cell death, which promotes a pro-inflammatory microenvironment. Some lesions evolve into vulnerable/unstable plaques characterized by necrotic areas filled with dead foam cells, low collagen content, and thin fibrous caps. This can lead to plaque rupture, precipitating acute, life-threatening cardiovascular events (22). Since ORP2 facilitates cholesterol transport to the plasma membrane, and previous research in A431 cells has shown that ORP2 mediates the flux of LDL-derived cholesterol from late endosomes/lysosomes (LE/Lys) to recycling endosomes (13, 15), ORP2 might play a role in cholesterol deposition within atherosclerotic lesions. Cholesterol crystals, major components of the atherosclerotic core, contribute to mechanical damage, cytotoxicity, and pro-inflammatory effects associated with lesion progression and rupture (23). Using polarization microscopy, we observed birefringent crystals in atherosclerotic lesions (24) and found a significant reduction in these crystals in the ORP2 overexpression group (Fig. 4*B*), indicating a decrease in cholesterol crystals. Additionally, ORP2 overexpression reduced neutral lipid deposition, necrotic core size, and macrophage content in atherosclerotic lesions, ultimately improving plaque stability.

Lipid metabolism in macrophages involves three key processes: cholesterol uptake, esterification, and efflux. Imbalances in these pathways lead to cholesterol accumulation and the formation of foam cells, which contribute to atherosclerotic plaque development (1, 3). Our study found that overexpression of ORP2 significantly inhibited foam cell formation. We then examined the expression of key proteins involved in lipid uptake, esterification, and efflux pathways. The results showed no significant changes in cholesterol uptake and esterification-related proteins, while the cholesterol efflux-related proteins LXRα and ABCA1/ABCG1 were significantly upregulated in hORP2^MOE^ PMCs. We also observed a significant increase in cholesterol efflux rate using the NBD-cholesterol assay, suggesting that ORP2 promotes macrophage cholesterol efflux. Our previous studies have demonstrated that ORP2 can facilitate cholesterol transport to the plasma membrane (13). Therefore, ORP2 may promote cholesterol efflux by increasing the accessible cholesterol content at the plasma membrane. We further confirmed this by detecting increased plasma membrane cholesterol in ORP2-overexpressing cells using D4_YDA_ probes (25).

LXRs are ligand-activated transcription factors belonging to the nuclear receptor superfamily. There are two subtypes, LXRα and LXRβ, encoded by genes *Nr1h3* and *Nr1h2*, respectively (26). While LXRα and LXRβ share high sequence homology, they differ in tissue distribution. LXRα is highly expressed in metabolically active tissues such as the intestine, liver, macrophages, and adipose tissue, whereas LXRβ is more widely expressed across tissues (27). In macrophages, LXRs promote cholesterol efflux and reduce lipid accumulation by activating downstream target genes like ABCA1 and ABCG1. Notably, overexpression of LXRα in macrophages has been shown to significantly reduce the development of atherosclerotic lesions (28, 29, 30). Previous studies have shown that ORP2 interacts with LXRα in the H295R adrenal cortex cell line, facilitating its nuclear translocation and transcriptional activation of the ABCA1 gene (21). However, these findings were primarily observed in non-macrophage cells, and the functional consequences of ORP2-LXRα interaction in macrophages under atherosclerotic conditions remained unknown. In this study, we investigated the relationship between ORP2 and LXRα in macrophages using hORP2 overexpressing PMCs. Through co-immunoprecipitation, immunofluorescence, and cytoplasmic/nuclear fractionation experiments, we confirmed that ORP2 interacts with LXRα and promotes nuclear localization. To further assess the impact of ORP2 on macrophage foam cell formation and cholesterol homeostasis *via* the LXRα pathway, we treated hORP2^MOE^ PMCs with the LXR antagonist GSK2033. Our results demonstrated that inhibiting LXR blocked the decrease in foam cell formation and the increase in LXRα nuclear translocation induced by ORP2 overexpression. These results underscore the importance of the ORP2–LXRα axis in modulating macrophage lipid metabolism in the context of atherosclerosis.

However, there are several limitations to our study. First, the effect of the LXRα antagonist on atherosclerotic plaque development was not evaluated *in vivo* in the ORP2 overexpressing mouse model. Second, the use of genetically modified mice that overexpress ORP2 in myeloid cells results in non-physiologically high levels of ORP2, which may not accurately reflect its endogenous function under pathological conditions. Thus, while this study provides important mechanistic insights, the relevance and quantitative impact of endogenous ORP2 in atherosclerosis remain to be fully established. Future studies employing myeloid-specific ORP2 knockout models will be essential to address this knowledge gap and validate the physiological role of ORP2. Additionally, if ORP2 is to be considered as a therapeutic target, viral gene delivery systems such as adeno-associated virus (AAV)-mediated overexpression may serve as a more translational and clinically feasible strategy compared to germline transgenics. These approaches would enable temporal and dosage-controlled modulation of ORP2, better simulating therapeutic scenarios.

In conclusion, our study provides the first *in vivo* evidence that ORP2 plays a protective role in macrophage cholesterol metabolism and atherosclerosis by promoting cholesterol efflux *via* two mechanisms: increasing plasma membrane cholesterol and facilitating the nuclear translocation of LXRα. This research highlights the physical and functional interactions between ORP2 and LXRα in regulating macrophage cholesterol homeostasis and offers new insights into potential therapeutic strategies for treating cardiovascular diseases.

## Experimental procedures

### Animals

All the animals were bred on a C57BL/6J (Vital River) background and housed under specific pathogen-free conditions in Hebei Medical University, and maintained on a 12 h light/12 h dark cycle at 24 °C, with free access to water and diet. The hORP2^flox/flox^ mice were generated by Cyagen Biosciences. LysM^cre^ transgenic mice were obtained from Jackson Laboratory (Stock No. 004781). ApoE^−/−^ mice were obtained from Vital River Laboratory. Myeloid-specific human ORP2 overexpression (hORP2^MOE^) mice were generated by crossing hORP2^flox/flox^ with LysM^cre^ transgenic mice. All LysM^cre^ mice used were heterozygous (Cre^+/−^) to preserve endogenous LysM gene function and avoid any confounding effects due to LysM knockout. For breeding, hORP2^flox/flox^ homozygous mice that were also heterozygous for the LysM-Cre transgene (Cre^+/−^) were bred with hORP2^flox/flox^ mice lacking the Cre transgene (Cre^−/−^) This breeding strategy ensured that all offspring carried homozygous hORP2^flox/flox^ alleles, while 50% of the progeny were Cre^+/−^ (designated as hORP2^MOE^) and the remaining 50% were Cre^−/−^, serving as littermate controls (hORP2^f/f^). Myeloid-specific ORP2 overexpression mice in ApoE^−/−^ background were generated by crossing hORP2^MOE^ mice with ApoE^−/−^ mice.

To investigate diet-induced hyperlipidemia and atherosclerosis, 10 to 12-week-old male hORP2^MOE^/ApoE^−/−^ and control mice were fed with a Western-type high-fat diet (HFD) containing 0.15% cholesterol and 20% lard fat for 12 or 18 weeks, respectively. Mice were anesthetized with 1% pentobarbital sodium and euthanized by Cervical dislocation. All procedures were followed to the NIH Guide for the Care and Use of Laboratory Animals (NIH publication no.85Y23, revised 1996) and approved by the Laboratory Animal Ethical and Welfare Committee of Hebei Medical University (IACUC-Hebmu-P 2022035).

### Cell culture

RAW264.7 cells (ATCC, Cat#TIB-71) and HEK293T (ATCC, Cat#Delf-10618) cells were cultured in Dulbecco’s Modified Eagle’s Medium (DMEM) containing 10% heat-inactivated fetal bovine serum (FBS), 100 units/ml penicillin, and 100 μg/ml streptomycin in a humidified atmosphere of 5% CO_2_ at 37 °C. Human monocyte line THP-1 cells (HyCyte) were cultured in RPMI-1640 medium containing 10% FBS, 100 units/ml penicillin, and 100 μg/ml streptomycin. Primary peritoneal macrophages (PMCs) were collected from peritoneal exudates 3 days after injecting mice with 2 ml of 4% BBL thioglycollate medium brewer modified (BD), and then cultured in RPMI-1640 supplemented with 10% FBS, 100 units/ml penicillin, and 100 μg/ml streptomycin.

### Foam cell formation assay

Macrophages were incubated with 25 μg/ml ox-LDL (Yiyuan Biotechnology, Guangzhou, CHN) for 24 h. After washing with PBS, cells were fixed with 4% paraformaldehyde for 10 min. Then, cells were incubated with 60% isopropanol for 5 min, stained with 0.5% Oil Red O (Sigma) in isopropanol for 30 min, then washed with 60% isopropanol. After washing with water, Hematoxylin staining was performed. Macrophages were photographed under a microscope at 400 × magnifications.

### Cholesterol efflux assay

Cholesterol efflux rate was measured as previous described (31). Briefly, macrophages were labeled with 5 μM NBD-cholesterol (Invitrogen) overnight and then washed 3 times with PBS. The medium was replaced with serum-free medium with or without ApoA-I (Milipore) (10 μg/ml) and incubated at 37 °C for 6 h. The medium was then collected and the fluorescence intensity (FI) at the 463/536 nm (excitation/emission) wavelength was further measured in a luminometer (MD SpectraMax i3). The cells were dissolved in the cell lysis solution (50 mM Tris-Cl, pH8.0, 150 mM NaCl, Triton-100/NP-40%) for 1 h, then FI at 463/536 nm(excitation/emission) wavelength was measured in a luminometer. The rate of cholesterol efflux was expressed as % cholesterol efflux, calculated as [medium fluorescence/(medium + cell lysate fluorescence)] × 100%.

### D4_YDA_ staining

To visualize cholesterol in cellular membranes, domain 4 (D4) of Perfringolysin O (PFO, theta toxin), a cholesterol-binding toxin was used as a biosensor. To increase the cholesterol affinity of the D4, three amino acids in the D4 were mutated (Y415A, D434W, and A463W) (25). The D4_YDA_ cDNA was introduced into a pLVX-mCherry-N1 vector at the EcoRI/BamHI site to construct mCherry-D4_YDA_ plasmid. For plasma membrane cholesterol staining, the mCherry-D4_YDA_ plasmid was transiently transfected into HEK293T cells using Hiperfect Transfection Reagent (TransIntro) according to the manufacturer’s instructions and was visualized by fluorescence photography (Leica TCS SP5).

### Cell immunofluorescence staining

PMCs or HEK293T cells were grown on glass coverslips and fixed in 4% paraformaldehyde for 30 min, and then permeabilized with 0.1 to 0.5% Triton X-100 at room temperature for 20 min. After that, the cells were blocked with 5% goat serum albumin in PBS for 1 h and incubated with primary antibodies at 4 °C overnight. Samples were washed 3 times with PBS for 15 min and incubated with corresponding fluorescent secondary antibodies for 1 h at room temperature. After washing in PBS for 3 times, nuclei were stained with DAPI. Confocal imaging was performed using a Leica TCS SP5 microscope equipped with a 63× oil immersion objective (Leica Microsystems). The following primary antibodies were used: anti-LXRα (BOSTER), anti-Flag (MBL). Co-occurrence of fluorescence signal was determined by direct line analysis measurement in Image J.

### Western blot and immunoprecipitation

Western blot analysis was performed as described previously (32). In brief, protein extracts were prepared from isolated cells or tissue by using lysis buffer containing a protease inhibitor cocktail and quantified using a BCA Assay Kit (Seven). Lysates containing equal amounts of total protein were subjected to 6%–12% sodium dodecyl sulfate-polyacrylamide gel electrophoresis and transferred onto polyvinylidene fluoride membranes. The membranes were blocked using a blocking reagent (Tris-buffered saline with 5% [w/v] non-fat dry milk and 0.1% [v/v] Tween 20, pH 7.4) for 1 h at room temperature and then incubated with the primary antibodies at 4 °C overnight. After incubation with the secondary HRP-linked anti-mouse IgG or HRP-linked anti-rabbit IgG antibody for 1 h at room temperature, the blots were visualized using an ECL kit and quantified using the ImageJ software (National Institutes of Health). For quantification, band intensity was background-subtracted and normalized to loading controls. The following primary antibodies were used for immunoblotting: anti-ORP2 (Abcam), anti-*β*-actin (Abclonal), anti-SRA1 (Proteintech), anti-CD36 (Abclonal). anti-SOAT1 (AbclonalN), anti-LXRα (BOSTER), anti-ABCA1 (Abclonal), anti-ABCG1 (Abclonal), and anti-Histone H3 (Servicebio). Based on the manufacturer’s instructions, the molecular weights of the target proteins, and supporting literature, we confirmed that the observed bands correspond to the expected target proteins.

For the immunoprecipitation assay, cell lysates were adjusted to equal amounts of total protein (500 μg) and incubated with the Flag antibody or control rMouse IgG (Abclonal) antibody overnight at 4 °C, followed by immunoprecipitation using the Protein PLUS A/G Agarose (Santa Cruz Biotechnology). After several washes with ice-cold PBS, the immunoprecipitates were subjected to western blotting as described above.

### Quantitative real-time PCR

Total RNA was extracted using TRIzol reagent (TIANGEN), and the cDNA was synthesized using cDNA Synthesis Kit (Servicebio). Real-time PCR was performed using the SYBR Green PCR Master Mix (Servicebio) on a QuantStudio Real-time PCR Detection System. Individual real-time quantitative PCR was performed using gene-specific primers as shown in Table S1. All relative gene expression levels were normalized to *Gapdh*.

### Blood biochemical analysis

Blood samples were collected through the retro-orbital vein from indicated animals fasted for 12 h. The levels of plasma total cholesterol, triglyceride, and glucose were determined by using enzymatic methods (Bio Sino). High-density lipoprotein cholesterol (HDL-C) level was measured with TC kit after precipitating apoB-containing lipoprotein by 20% polyethylene glycol (PEG).

### Quantification of atherosclerotic lesions

After 12 or 18 weeks of high-fat diet feeding, the mice were anesthetized with 1% pentobarbital sodium, euthanized, and then perfused with PBS through the left ventricle. Heart and aortic tissues were removed from the ascending aorta to the iliac artery bifurcation and fixed with 4% paraformaldehyde. To analyze the lesion area in the aortic root, the heart was dissected from the aorta, embedded in OCT compound, snap-frozen in liquid nitrogen and stored at −20 °C prior to sectioning. Serial 8-μm sections were obtained from the origin of the aorta where all three aortic valve cusps became clearly visible. For the analysis of atherosclerotic lesions, lipid deposition in the whole aorta (*en face*) and aortic root was examined by staining with 0.5% Oil Red O solution and hematoxylin. Oil Red O positive areas were quantified by Image J for atherosclerotic lesions. For the analysis of atherosclerotic plaque composition in aortic roots, cholesterol crystals from frozen sections were observed using a polarized light microscope (SOPTO CX40P). Immuno-detection was performed with CD68 antibody (Servicebio) for macrophage. Sections were also stained with Sirius Red (Solarbio) for fibrosis analysis.

### Statistical analysis

Data were presented as mean ± SD. Statistical analyses were performed using GraphPad Prism 8 software. Data were first tested for a normal distribution using the Shapiro–Wilk test. The significance of differences between two groups was assessed using Student's *t* test (for normally distributed data) or the Wilcoxon rank-sum test (for non-normally distributed data). For comparisons involving more than two groups, one-way ANOVA followed by Tukey's multiple comparison test was used. When comparing multiple groups with more than two variables, a two-way ANOVA was performed, followed by Sidak’s multiple comparison test. A *p*-value of <0.05 was considered statistically significant.

Additional information on key reagents and resources can be found in the Table S2.

## Compliance with ethics requirements

The experimental design and protocols were approved by the Laboratory Animal Ethical and Welfare Committee of Hebei Medical University (IACUC-Hebmu-P 2022035) and were also complied with the Guidelines for Care and Use of Laboratory Animals published by the US National Institutes of Health.

## Data availability

All data generated or analyzed during this study are included in this published article and its supplementary information files.

## Supporting information

This article contains supporting information.

## Conflict of interest

The authors declare that they have no conflicts of interest with the contents of this article.
