# Supplementary material for: The macrophage sterol transport protein ORP2 promotes cholesterol efflux and prevents foam cell formation and atherosclerosis
Source: J Biol Chem. 2025 May 9;301(6):110228. doi: 10.1016/j.jbc.2025.110228 (PMC12167482; doi:10.1016/j.jbc.2025.110228)
Supplement: Supporting information [file mmc1.docx]

Macrophage ORP2 promotes cholesterol efflux and prevents foam cell formation and atherosclerosis

*Running title:* ORP2 prevents Atherosclerosis

Xiaowei Wang, Kenan Peng, Yudi Zhao, Liwen Qiu, Chenxi Liang, Yaqian Dou, Qianqian Dong, Xiaoting Ma, Jinye Tang, Yidan Ma, Lin Liu, Mingqi Zheng, Hongyuan Yang, Mingming Gao

**Supplementary Figure S1. Construction of myeloid-specific hORP2 overexpression mice.**


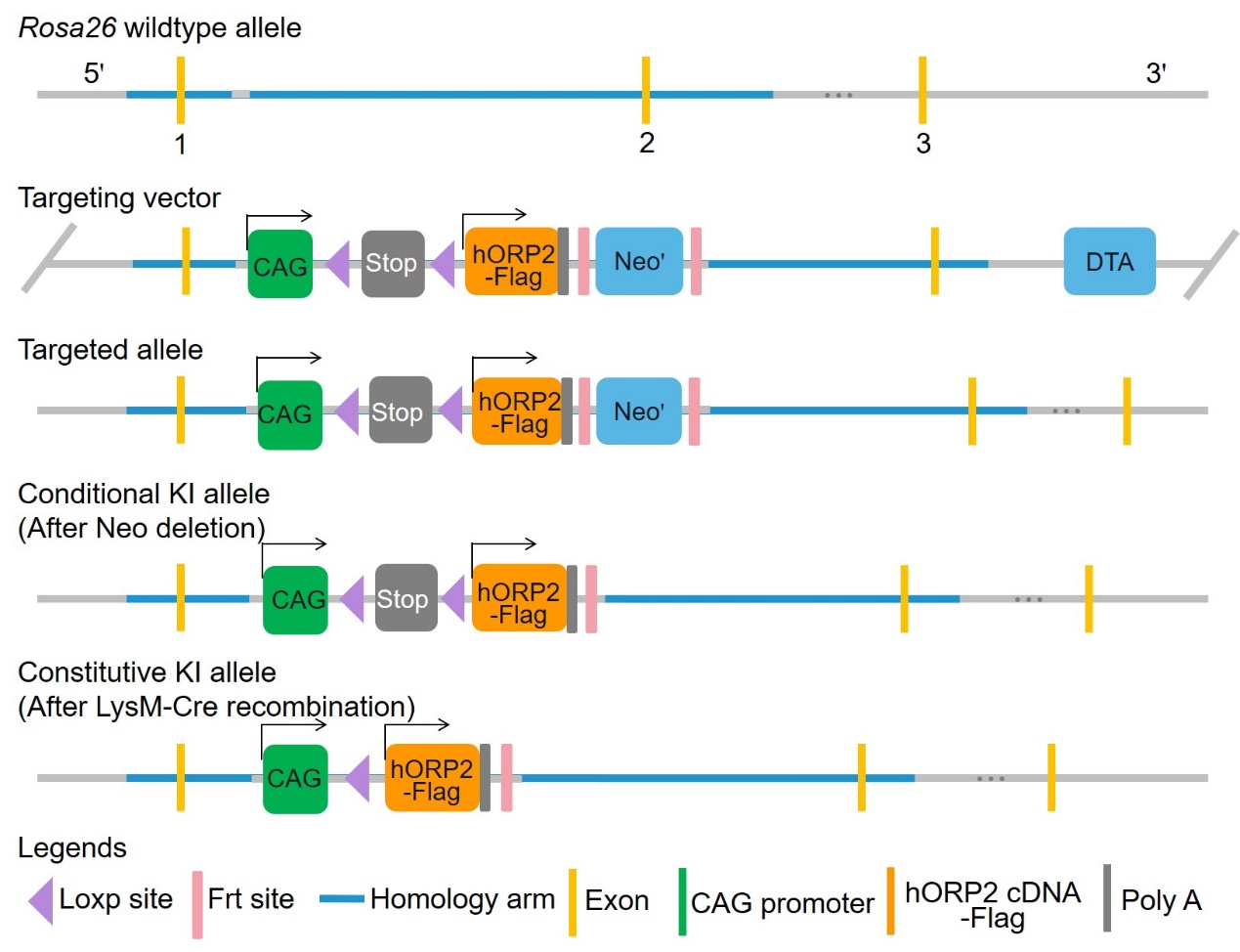


**Supplementary Figure S2. Related to Figure 5A and 6D.**


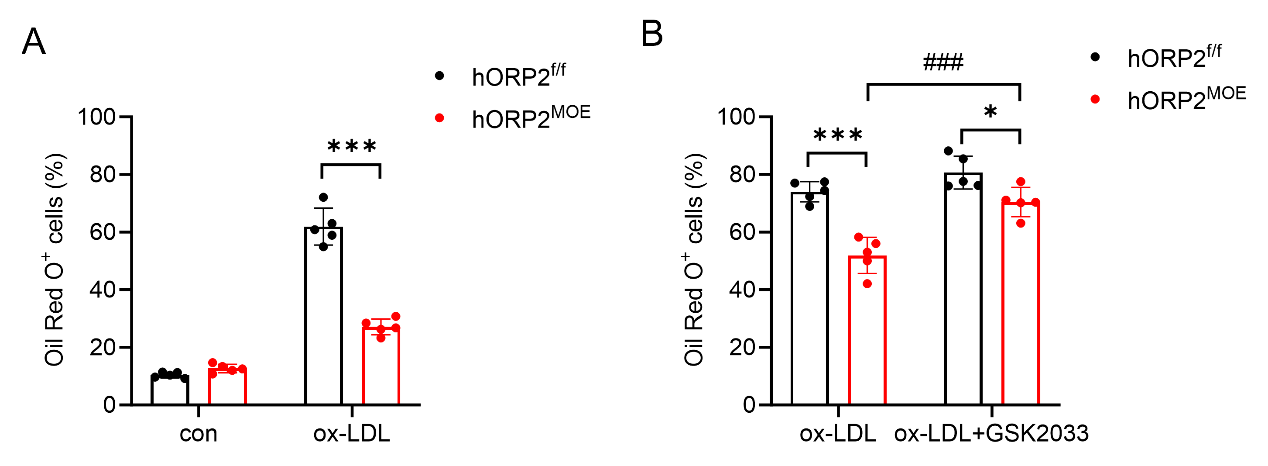


**Supplementary Figure S2. Related to Figure 5A and 6D.**

1. Quantification of Oil Red O positive cells of Figure 5A (n = 5 mice per group).
2. Quantification of Oil Red O positive cells of Figure 6D (n = 5 mice per group).

Data are presented as mean ± SD. Statistical significance was determined by two-way ANOVA with Tukey's multiple comparison test. **P*<0.05, ***/### *P* < 0.001.

**Supplementary Table S1. Real time PCR primer information.**

| Gene | Sense strand (5′-3′) | Antisense strand (5′-3′) | |
| --- | --- | --- | --- |
| mOsbpl2 | CCATGTTCACCAGAAGCGACT | | TTTGCCAGTTCTCTCCCACTG |
| Osbpl2 | GTTCTGGGGCAAAAGCGTG | | TCTCCACTGTCCCATACTGCT |
| Msr1 | TGGACCCCAAGGTGAAAAGG | | TCTTAAAAGGGTCTTGCCCCA |
| Cd36 | ATGGGCTGTGATCGGAACTG | | TTTGCCACGTCATCTGGGTTT |
| Pparg | AGGTCAGAGTCGCCCCG | | CTTGTCGTCACACTCGGTCC |
| Soat1 | CAGGAAGTAAGATGCCTGGAAC | | TGCAGCAGTACCAAGTTTAGTG |
| Nr1h3 | CTCAATGCCTGATGTTTCTCCT | | TCCAACCCTATCCCTAAAGCAA |
| Nr1h2 | ATGTCTTCCCCCACAAGTTCT | | GACCACGATGTAGGCAGAGC |
| Abca1 | AAAACCGCAGACATCCTTCAG | | CATACCGAAACTCGTTCACCC |
| Abcg1 | GTGGATGAGGTTGAGACAGACC | | CCTCGGGTACAGAGTAGGAAAG |
| Tnf | ACCCTCACACTCACAAACCA | | ATAGCAAATCGGCTGACGGT |
| Vcam1 | CTGGGAAGCTGGAACGAAGT | | GCCAAACACTTGACCGTGAC |
| Il1b | TGCCACCTTTTGACAGTGATG | | AAGGTCCACGGGAAAGACAC |
| Ccl2 | AGGTGTCCCAAAGAAGCTGT | | AAGACCTTAGGGCAGATGCAG |
| Arg1 | TTTTAGGGTTACGGCCGGTG | | TTTGAGAAAGGCGCTCCGAT |
| Il10 | ACTTGGGTTGCCAAGCCTTA | | GACACCTTGGTCTTGGAGCTTA |
| *Gapdh* | TGATGACATCAAGAAGGTGGTGAAG | | TCCTTGGAGGCCATGTAGGCCAT |

**Supplementary Table S2. Key resources table.**

| REAGENT or RESOURCE | SOURCE | IDENTIFIER |
| --- | --- | --- |
| Antibodies |  |  |
| Rabbit polyclonal anti-ORP2 | Abcam | Cat#ab235298;  RRID: AB_3083070 |
| Rabbit monoclonal anti-SRA1 | Proteintech | Cat#24655-1-AP;  RRID: AB_2879657 |
| Rabbit monoclonal anti-CD36 | Abclonal | Cat#A19016;  RRID: AB_2862508 |
| Rabbit monoclonal anti-SOAT1 | Abclonal | Cat#A13273;  RRID: AB_2760125 |
| Rabbit polyclonal anti-LXRα | BOSTER | Cat#BA2757-2; |
| Rabbit monoclonal anti-ABCA1 | Abclonal | Cat#A7228;  RRID: AB_2767776 |
| Rabbit monoclonal anti-ABCG1 | Abclonal | Cat#A4328;  RRID: AB_2765627 |
| Rabbit monoclonal anti-CD68 | Servicebio | Cat#GB113109;  RRID: AB_2935658 |
| Mouse monoclonal anti-IgG | Abclonal | Cat#AC011  RRID: AB_2770414 |
| Mouse monoclonal anti-Flag | MBL | Cat#M185-3L |
| Cy3 conjugated Goat Anti-Rabbit IgG | Servicebio | Cat#GB21303;  RRID: AB_2861435 |
| FITC conjugated Goat Anti-Mouse IgG | Servicebio | Cat#GB22301;  RRID: AB_3096857 |
| Rabbit monoclonal anti-Histone H3 | Servicebio | Cat#GB11102;  RRID: AB_3073627 |
| Rabbit monoclonal anti-GAPDH | Servicebio | Cat#GB11002;  RRID: AB_2904017 |
| Rabbit monoclonal anti-*β*-actin | Abclonal | Cat#AC026;  RRID: AB_2768234 |
| Chemicals |  |  |
| BBL thioglycollate medium brewer modified | BD | Cat#211716 |
| ox-LDL | Yiyuan Biotechnology | Cat#YB-002 |
| PMA | Sigma | Cat#16561-29-8 |
| NBD-cholesterol | Invitrogen | Cat#N1148 |
| Apolipoprotein A-Ⅰ | Milipore | Cat#ALP10 |
| LXR agonist T0901317 | Sigma | Cat#293754-55-9 |
| LXR antagonist GSK2033 | MCE | Cat#1221277-90-2 |
| Oil Red O | Sigma | Cat#1320-06-5 |
| Sirius Red | Solarbio | Cat#S8060 |
| Protein PLUS A/G Agarose | Santa Cruz Biotechnology | Cat#sc-2003 |
| CHO kit | Biosino | Cat#100000180 |
| TG kit | Biosino | Cat#100000220 |
| Glu kit | Biosino | Cat#100000240 |
| BCA Assay Kit | Seven | Cat#SW101 |
| ECL kit | Servicebio | Cat#G2014 |
| Nuclear and Cytoplasmic Protein Extraction Kit | Beyotime | Cat#P0028 |
| Cocktail | Servicebio | Cat#G2006 |
| TRIzol | TIANGEN | Cat#DP424 |
| cDNA Synthesis Kit | Servicebio | Cat#G3337 |
| SYBR Green PCR Master Mix | Servicebio | Cat#G3326 |
| Hiperfect Transfection Reagent | TransIntro | Cat#FT201 |
| Experimental models: Cell lines |  |  |
| RAW 264.7 | ATCC | Cat#TIB-71 |
| HEK293T | ATCC | Cat#Delf-10618 |
| THP1 | HyCyte | Cat#TCH-C361 |
| Experimental models: Organisms/ strains | | |
| C57BL/6J mice | Charles River | N/A |
| LyzM-Cre mice | Jackson laboratory | Stock No. 004781 |
| ApoE^-/-^ mice | Charles River | N/A |
| hORP2^flox/flox^ mice | This paper | N/A |
| ORP2^flox/flox^ mice | This paper | N/A |
| Recombinant DNA |  |  |
| hORP2 cloned onto a pEGFPc-1 vector | This paper | N/A |
| D4_YDA_ cloned onto a pLVX-mCherry-N1 vector | This paper | N/A |
| Software and algorithms |  |  |
| ImageJ | National Institutes of Health | https://ImageJ.net/software/ImageJ/ |
| GraphPad Prism 8.0 | GraphPad | https://www.graphpad.com/ |
| Adobe Photoshop CS6 | Adobe | https://www.adobe.com/ |
| Adobe Illustrator CS6 | Adobe | https://www.adobe.com/ |
